# Broadened Angle-Insensitive Near-Perfect Absorber Based on Mie Resonances in Amorphous Silicon Metasurface

**DOI:** 10.3390/nano10091733

**Published:** 2020-09-01

**Authors:** Jiangnan Si, Shuang Liu, Weiji Yang, Xuanyi Yu, Jialin Zhang, Xiaoxu Deng

**Affiliations:** State Key Laboratory of Advanced Optical Communication Systems and Networks, Key Laboratory for Laser Plasmas (Ministry of Education), School of Physics and Astronomy, Shanghai Jiao Tong University, Shanghai 200240, China; 1359231351370@sjtu.edu.cn (J.S.); 503313461@sjtu.edu.cn (S.L.); yangweiji@sjtu.edu.cn (W.Y.); yxy1593725@sjtu.edu.cn (X.Y.); dorismalfoy@sjtu.edu.cn (J.Z.)

**Keywords:** amorphous silicon metasurface, perfect absorption, Mie resonances

## Abstract

A broadband near-perfect absorber is analyzed by an amorphous silicon (a-Si) hook shaped nanostructure metasurface. The transmission and reflection coefficients of the metasurface are investigated in the point electric and magnetic dipole approximation. By combining square and semicircle nanostructures, the effective polarizabilities of the a-Si metasurface calculated based on discrete dipole approximation (DDA) exhibit broadened peaks of electric dipole (ED) and magnetic dipole (MD) Mie resonances. The optical spectra of the metasurface are simulated with different periods, in which suppressed transmission are shifted spectrally to overlap with each other, leading to broadened enhanced absorption induced by interference of ED and MD Mie resonances. The angle insensitive absorption of the metasurface arrives 95% in simulation and 85% in experiment in spectral range from 564 nm to 584 nm, which provides potential applicability in nano-photonic fields of energy harvesting and energy collection.

## 1. Introduction

Metasurfaces are artificial planar material composed of spatially arranged subwavelength structures, which exhibit remarkable flexibility in manipulating the properties of light at an optically thin interface [1,2,3]. Perfect absorbers based on metasurfaces for energy collection and accumulation have been widely researched, which are promising in applications of light harvesting [4,5,6,7,8], optical isolation [9,10] and so on. Dielectric nanostructure metasurfaces, holding advantages including nonradiative loss [11,12,13], resonant enhancement of both electric and magnetic field [14,15,16,17,18,19], attract much attention in research of high absorption induced by the Mie dipole resonances [20,21,22,23]. Recently, Länk’s group has proposed a silicon nano-pillar metasurface in a total internal reflection geometry to realize near-perfect absorption in near-infrared region resulting from interference between coherent electric and magnetic dipoles scattering as well as reflection from supporting glass substrate [15]. Yang’s group has reported a narrow band perfect absorber with a high quality factor by an amorphous silicon (a-Si) nano antenna metasurface, which is realized due to not only the coherent ED and MD Mie resonances but also the intrinsic loss of material [23]. Liu’s group has put forward a germanium moth-eye nanostructure to obtain a perfect selective absorption attributed by the electric field enhancement due to electric and magnetic multipole resonances, which is insensitive to incident angle from 0° to 75° [24].

In this manuscript, a dielectric broadband near-perfect absorber is numerically studied and experimentally analyzed based on an amorphous silicon (a-Si) hook shaped nanostructure metasurface. The transmission and reflection coefficients of the a-Si hook shaped nanostructure metasurface are investigated by far-field scattering of electric and magnetic dipole moments. The electric dipole (ED) and magnetic dipole (MD) effective polarizabilities of the a-Si nanostructure metasurface are numerically calculated from simulated displacement currents based on the discrete dipole approximation (DDA). ED and MD Mie resonances of the a-Si metasurface, which induce suppression band in the simulated transmission spectra, are periodical adjustable in spectral position. Due to the interference between overlapped Mie resonances, the absorption spectrum reaches the maximum of 95% in a broadened spectral range from 564 nm to 584 nm, which is induced by enhanced E-fields in different positions of the hook shaped nanostructure. Furthermore, the near perfect absorption is changeless with the incident angle varying from 0° to 20° due to angle insensitive Mie resonances. The nanoscale a-Si metasurface was fabricated and the absorption spectrum measured by a convergent spectrometer arrives 85% in the same resonant spectral range in simulation. The fabricated broadened band incident-angle-insensitive near-perfect absorber has promising prospects of practical nano-photonic applications in area of optical trapping, energy accumulation and energy harvesting.

## 2. Materials and Methods

**Figure 1:**
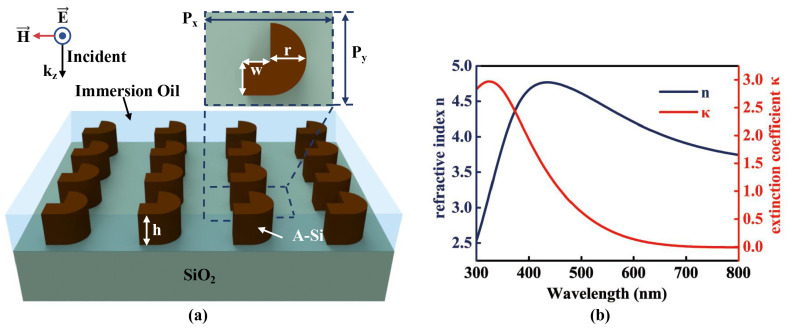
(**a**) The schematic of the a-Si hook shaped nanostructure metasurface. The unit cell is shown in the top figure. (**b**) Refractive index *n* (blue curve) and extinction coefficient κ (red curve) of amorphous silicon film.

The incident-angle insensitive dielectric broadband near-perfect absorber in this manuscript is an a-Si hook shaped nanostructure periodical array metasurface, of which the schematic is presented in Figure 1a. The unit cell of the metasurface contains a nano square and a nano semicircle, as shown in the inset of Figure 1a. The edge width of a square is *w* = 70 nm. The radius of a semicircle is *r* = 75 nm. Periods of the nanostructure metasurface in the x and y direction are $P_{x}$ = 220 nm and $P_{y}$ = 340 nm, respectively. The a-Si nanostructure thickness *h* is 115 nm. The hook shaped nanostructure metasurface is on the top of SiO${}_{2}$ substrate and submerged in immersion oil. The refractive indexes of SiO${}_{2}$ and immersion oil are $n_{SiO_{2}}$ = 1.528 and $n_{Oil}$ = 1.485, respectively. The three-dimensional finite-difference time-domain method (FDTD) is utilized to simulate the optical spectra and the electric fields of the a-Si metasurface. The boundary condition in FDTD simulation is periodic in the x and y-directions, and the perfect matched layer (PML) in the z-direction. A plane wave is set in the FDTD simulation, of which the wave vector is along the negative z direction, the electric field is parallel to the x direction and the magnetic field is parallel to the y direction. The reflection (*R*), transmission (*T*) and the absorption (*A*) of the a-Si metasurface and the displacement currents of the a-Si hook shaped nanostructure are simulated by FDTD.

In the fabrication process, the a-Si film was deposited on the SiO${}_{2}$ substrate by Plasma Enhanced Chemical Vapor Deposition system. The complex refractive index of the a-Si is *n*${}_{a-Si}$ = *n* + *i*κ, where the real part is defined as the refractive index *n* and the imaginary part is defined as the extinction coefficient κ. *n* and κ are measured by spectroscopic ellipsometry apparatus from the fabricated a-Si film, as presented in Figure 1b. Photoresist was first coated on the a-Si film, then 20 nm thick aluminum film was deposited on the photoresist as a conducting layer. The a-Si hook shaped nanostructure metasurface was fabricated by electron-beam lithography (EBL) and the silicon etching system. Finally, the immersion oil (*n* = 1.485) was added from the edge of the fabricated metasurface and submerge the nanostructures.

## 3. Results and Discussion

### 3.1. Numerical and Simulated Results

The broadband Mie resonances of the a-Si hook shaped nanostructure metasurface are investigated by effective polarizabilities based on discrete dipole approximation (DDA). The light-induced electric dipole (ED) and magnetic dipole (MD) moment of an a-Si hook shaped nanostructure in the periodical array are related with displacement currents [25]:

(1) $$p=-\frac{1}{i\omega }\int Jd^{3}r;\;m=\int \left(r\times J\right)d^{3}r$$

where ω is the frequency of incident light, *J* is the displacement current. Under illumination of x-polarized light, only the ED moment $p_{x}$ and the MD moment $m_{y}$ of the a-Si hook shaped nanostructure contribute to the light scattering in the far-field approach due to the periodicity of the metasurface [26], while the scattered light contributed by $m_{z}$ is ignored. Every a-Si hook shaped nanostructure are seen as point sources with same effective ED polarizability $\alpha_{eff}^{E}=p_{x}/\varepsilon_{0}E^{0}$ and MD polarizability $\alpha_{eff}^{M}=m_{y}/H^{0}$, where $E^{0}$ and $H^{0}$ are the electric field and magnetic field of the x-polarized light illuminated on the periodical array, respectively, and $\varepsilon_{0}$ is dielectric constant of vacuum. Based on the scattered fields generated by the point source dipole moments of the a-Si hook shaped nanostructure in the far-field approach, the transmission (*t*) and reflection (*r*) coefficients of the periodical array are:

(2) $$t=1+\frac{ik_{0}}{2S_{L}}\left(\alpha_{eff}^{E}+\alpha_{eff}^{H}\right),\;r=\frac{ik_{0}}{2S_{L}}\left(\alpha_{eff}^{E}-\alpha_{eff}^{H}\right)$$

$k_{0}$ is the wave number; $S_{L}$ is the unit cell area. According to Equation (2), the transmission and reflection coefficients simultaneously approach zero when:

(3) $$Re\left(\alpha_{eff}^{E}\right)=Re\left(\alpha_{eff}^{H}\right)=0,\;Im\left(\alpha_{eff}^{E}\right)=Im\left(\alpha_{eff}^{H}\right)=S_{L}/k_{0}$$

**Figure 2:**
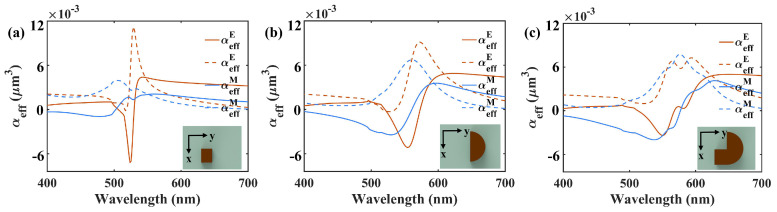
The effective polarizabilities of the square only nanostructure (**a**), the semicircle only nanostructure (**b**) and the hook shaped nanostructure (**c**) with periods $P_{x}$ = 220 nm, $P_{y}$ = 340 nm.

The effective polarizabilities of the square only nanostructure, the semicircle only nanostructure and the hook shaped nanostructure metasurfaces are calculated by utilizing FDTD-simulated displacement currents in spectral range from 400 nm to 800 nm with $P_{x}$ = 220 nm and $P_{y}$ = 340 nm based on DDA, as shown in Figure 2. The ED and MD effective polarizabilities of the square only nanostructure metasurface are shown by the red and blue curves in Figure 2a, respectively. $Im(\alpha_{eff}^{E})$ has a sharp peak and $Im(\alpha_{eff}^{M})$ possesses a low peak on behalf of the ED and MD resonances, respectively, which are at spectral positions of 540 nm and 500 nm where the corresponding real parts of effective polarizabilties are around zero. For the semicircle only nanostructure metasurface, $Im(\alpha_{eff}^{E})$ and $Im(\alpha_{eff}^{M})$ hold peaks with different value at spectral positions of 580 nm and 570 nm, respectively, as shown by red and blue dashed curves in Figure 2b. With respect to the hook shaped nanostructure metasurface, $Im(\alpha_{eff}^{E})$ and $Im(\alpha_{eff}^{M})$ both have a broadened peak approximately equal to $S_{L}/k_{0}$ in spectral range from 564 nm to 584 nm, meanwhile $Re(\alpha_{eff}^{E})$ and $Re(\alpha_{eff}^{M})$ are around zero, as shown in Figure 2c. The ED and MD Mie resonances of the metasurface are both broadened due to the employed square and semicircle in a hook shaped nanostructure.

**Figure 3:**
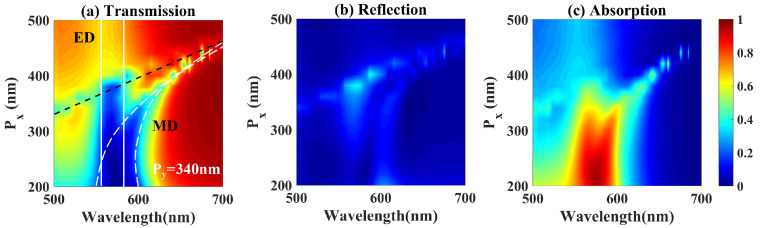
The simulated (**a**) transmission, (**b**) reflection, and (**c**) absorption spectra of the metasurface with different periods $P_{x}$. $P_{y}$ is fixed at 340 nm.

The impact of period on optical spectra of the a-Si hook shaped nanostructure metasurface is simulated by FDTD. The simulated transmission (*T*), reflectance (*R*) and absorption (*A*) spectra of the a-Si hook shaped nanostructure metasurface with varying period $P_{x}$ and fixed $P_{y}$ = 340 nm are shown in Figure 3a–c, respectively. Two broadened suppressed transmission, one red shifted with *P*${}_{x}$, the other fixed around 570 nm exist in Figure 3a, which are induced by MD and ED resonances, respectively. When *P*${}_{x}$≈ 220 nm, in spectral range from 564 nm to 584 nm, reflection is strongly suppressed, meanwhile absorption approaches the maximum of 95%, as presented in Figure 3b,c, respectively. The interference of overlapped ED and MD resonances results in suppressed transmission by the electric field localization and suppressed reflection by the Kerker effect, therefore the broadband absorption enhancement of the a-Si hook shaped nanostructure metasurface is achieved.

**Figure 4:**
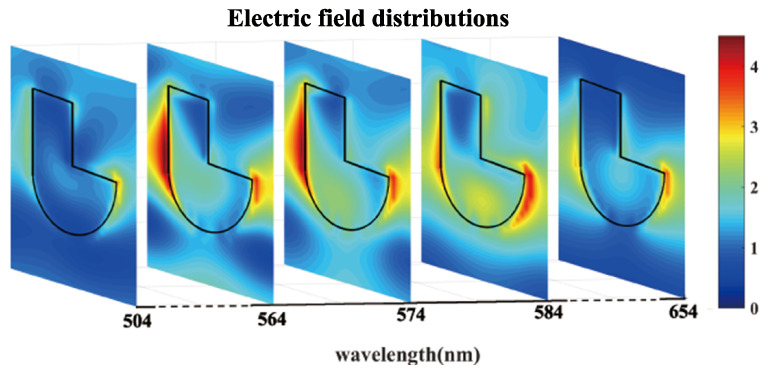
The electric field (E-field) distributions of the a-Si hook shaped nanostructure in the x-y plane when electric dipole (ED) and magnetic dipole (MD) resonances are overlapped at wavelength of 504 nm, 564 nm, 574 nm, 584 nm and 654 nm.

The electric field (E-field) distributions inside the a-Si hook shaped nanostructure metasurface at different wavelengths are simulated and analyzed. The E-field distributions of the hook shaped nanostructure in the x-y plane in the overlapped spectral range of ED and MD resonances from 564 nm to 584 nm are more intensive compared with those at non-resonant wavelengths of 504 nm and 654 nm, as presented in Figure 4. The enhancement of E-field distributions is induced in the square and semicircle nanostructure separately at different wavelengths, leading to the strengthened absorption in a broadened spectral range.

**Figure 5:**
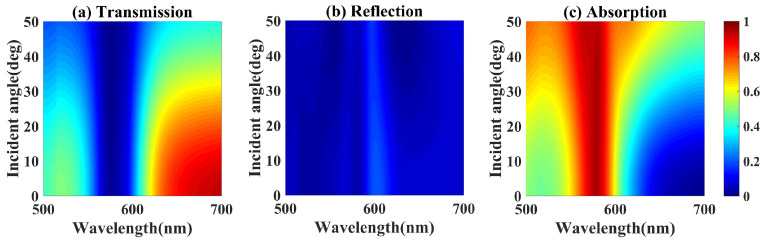
The simulated (**a**) reflection, (**b**) transmission and (**c**) absorption spectra of the a-Si nanostructure metasurface as a function of incident angle.

The optical spectra of the a-Si hook-shaped nanostructure metasurface are simulated with different incident angle θ. The simulated reflection, transmission and absorption spectra with incident angle θ from 0° to 50° in spectral range from 500 nm to 700 nm are presented in Figure 5a–c, respectively. The suppression band and suppression degree of both reflection and transmission are not affected by the incident angle increasing from 0° to 20°, therefore the enhanced absorption is unchanged in both spectral region and maximum value. When the incident angle increases from 20° to 50°, the suppression band of reflection is narrowed down, and the suppressed transmission is broadened and increased less than 3%, therefore the enhanced absorption of the metasurface is broadened and declined below 3%. The near-perfect absorption of the a-Si hook shaped nanostructure metasurface is efficient with incident angle varing from 0° to 20° due to incident angle insensitive ED and MD Mie resonances.

### 3.2. Experimental Results

**Figure 6:**
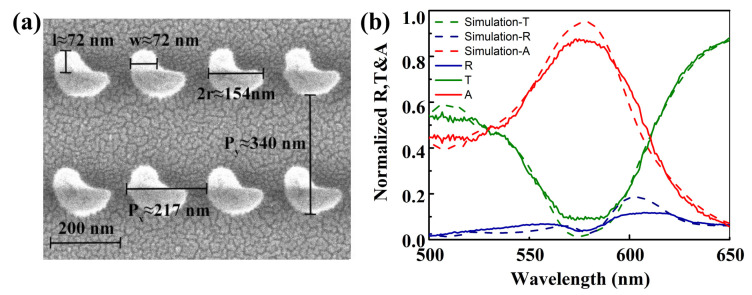
(**a**) The scanning electron microscopy (SEM) image of fabricated a-Si hook shaped nanostructure metasurface. (**b**) The measured reflection (*R*, blu solid curve), transmission (*T*, green solid curve) and absorption (*A*, red solid curve) spectra of the fabricated metasurface. The simulated reflection, transmission and absorption of the metasurface are presented by the dashed curves.

The scanning electron microscopy (SEM) image of the fabricated a-Si hook shaped nanostructure metasurface is presented in Figure 6a, in which the radius of a semicircle is *r* ≈ 77 nm and the edge length of the square is *w* ≈ 72 nm. Periods of the fabricated metasurface are $P_{x}$≈ 217 nm, $P_{y}$≈ 340 nm. Total pattern area of the fabricated metasurface is 499.4 μm × 499.8 μm. The thickness of the fabricated a-Si film was 114.8 nm. Transmission (*T*), reflection (*R*) and absorption (*A*) spectra of the fabricated a-Si metasurface were measured by a convergent spectrometer, as presented by the green, blue and red solid curves in Figure 6b, respectively, which exhibit same trend as simulated results shown by the dashed curves. The near-perfect absorption in experiment reaches 85% in the spectral range from 564 nm to 584 nm, which declines 10% compared with simulated result. The reduction of the measured near-perfect absorption is caused by the nano fabrication deviation of shape of the a-Si nanostructures, while the impact of convergent spectrometer on the measured spectra is neglected due to the incident angle insensitivity of the a-Si metasurface. The metasurface is not completely submerged by the immersion oil, leading to residual air gaps between the hook shaped nanostructures which also results in the reduction of measured near-perfect absorption.

## 4. Conclusions

In conclusion, a dielectric broadband near-perfect absorber based on amorphous silicon hook shaped nanostructure metasurface is investigated numerically and experimentally. The transmission and reflection coefficients of the a-Si metasurface are analyzed by far-field scattering of electric and magnetic dipole moments. By researching effective polarizabilities numerically calculated based on DDA, ED and MD Mie resonances of the a-Si metasurface are broadened in spectrum due to the employed hook shaped nanostructure. Angle insensitive near-perfect absorption of the a-Si metasurface reaches 95% in simulation and 85% in experiment in a broadened spectral range from 564 nm to 584 nm by the interference of Mie resonances. The proposed a-Si hook shaped nanostructure metasurface with characteristics of broadened bandwidth and insensitivity of incident angle, is prospective in practical applications including optical isolation device and energy harvesting system.
